# A case of bronchial Dieulafoy disease and literature review

**DOI:** 10.1186/s13019-023-02279-1

**Published:** 2023-06-27

**Authors:** Xiaoqian Shi, Mingdong Wang, Yifei Wang, Wei Zhang, Xuewei Zhao, Bing Li

**Affiliations:** 1 Department of Pulmonary and Critical Care Medicine, Shanghai Fourth People’s Hospital, School of Medicine, Tongji University, No. 1279 Sanmen Road, Shanghai, 200434 China; 2 Department of Thoracic Surgery, Shanghai Fourth People’s Hospital, School of Medicine, Tongji University, No. 1279 Sanmen Road, Shanghai, 200434 China; 3 Department of Pathology, Shanghai Fourth People’s Hospital, School of Medicine, Tongji University, No. 1279 Sanmen Road, Shanghai, 200434 China

**Keywords:** Dieulafoy disease of bronchus, Hemoptysis, Vascular malformation, Bronchial angiography, Case report

## Abstract

**Objective:**

Bronchial Dieulafoy's disease (BDD) is a rare disease that causes massive hemoptysis. This paper reports a case of BDD treated surgically. At the same time, we summarize the data of BDD patients reported in domestic and foreign literature to improve the understanding, diagnosis and treatment of this disease.

**Methods:**

A case of BDD with hemoptysis during bronchoscopy was reported. In addition, we searched for "bronchial Dieulafoy disease" through Pubmed, Web of Science, CNKI and Wanfang databases, covering the literature related to BDD that was definitely diagnosed or highly suspected from January 1995 to December 2021, and summarized the clinical characteristics, chest imaging, bronchoscopic manifestations, angiographic characteristics, pathological characteristics, treatment and outcome of patients.

**Results:**

The patient was a 68 year old male. Tracheoscopy revealed nodular and mass like changes in the basal segment of the left lower lobe, which appeared massive hemorrhage when touching the surface. The computed tomography angiophy of the bronchial artery confirmed that the branches of the left bronchial artery were tortuous and dilated, and then the left lower lobe of the lung was resected. During the operation, 3 thick tortuous nutrient artery vessels were sent out from the descending aorta, and 1 thick tortuous nutrient artery was sent out from the autonomic arch. All of them were ligated and cut. The pathology after the operation was in accordance with BDD; The patient did not have hemoptysis after discharge and is still under follow-up. The database identified 65 articles from January 1995 to December 2021. After removing repeated reports, meetings, incomplete information and nursing literature, 60 articles were included to report 88 cases of BDD. BDD can occur at all ages, with a male to female ratio of about 1.6:1. It mainly starts with hemoptysis, and can also be seen due to cough, infection, and respiratory failure; Inflammatory changes such as pulmonary patch shadow, exudation shadow and ground glass shadow of pulmonary hemorrhage were more common in chest imaging; The diagnosis of BDD is mainly based on the bronchoscopy, bronchial angiography and pathological findings of surgical or autopsy specimens. Bronchoscopic findings were mostly non pulsating, smooth nodular or mucosal processes. Bronchial angiography mainly showed tortuous dilatation of bronchial artery, and the lesions were mainly located in the right bronchus, more from the bronchial artery; Diagnosis depends on pathology, showing submucosal expansion of bronchus or abnormal artery rupture and bleeding; 54 cases underwent selective bronchial artery embolization, 39 cases underwent pulmonary lobectomy, 66 cases improved, and 10 cases died (all of them were caused by massive hemorrhage during bronchoscopic biopsy).

**Conclusion:**

BDD is rare, but may cause fatal massive hemoptysis. Bronchial angiography is considered to be an effective method to diagnose BDD. Since pathological biopsy may lead to fatal bleeding, the necessity of pathological diagnosis remains controversial. Interventional and surgical treatment plays an important role in patients with cough accompanied by massive hemoptysis.

## Introduction

Dieulafoy's disease was first reported by French doctor Georges Dieulafoy [1] in 1898. It often occurs in the gastrointestinal tract, also known as gastric submucosal aneurysm or Dieulafoy's ulcer. Dieulafoy disease of bronchus is rare, which is characterized by the presence of dysplastic arteries in the submucosa of bronchus, leading to artery expansion, distortion and rupture, and is prone to fatal bleeding. Since Sweets [2] first reported it in 1995, less than 100 cases of BDD have been reported. In order to improve the understanding of BDD and avoid fatal hemoptysis, this article describes the clinical characteristics, diagnosis and treatment of BDD.

## Case report

**Fig. 1:**
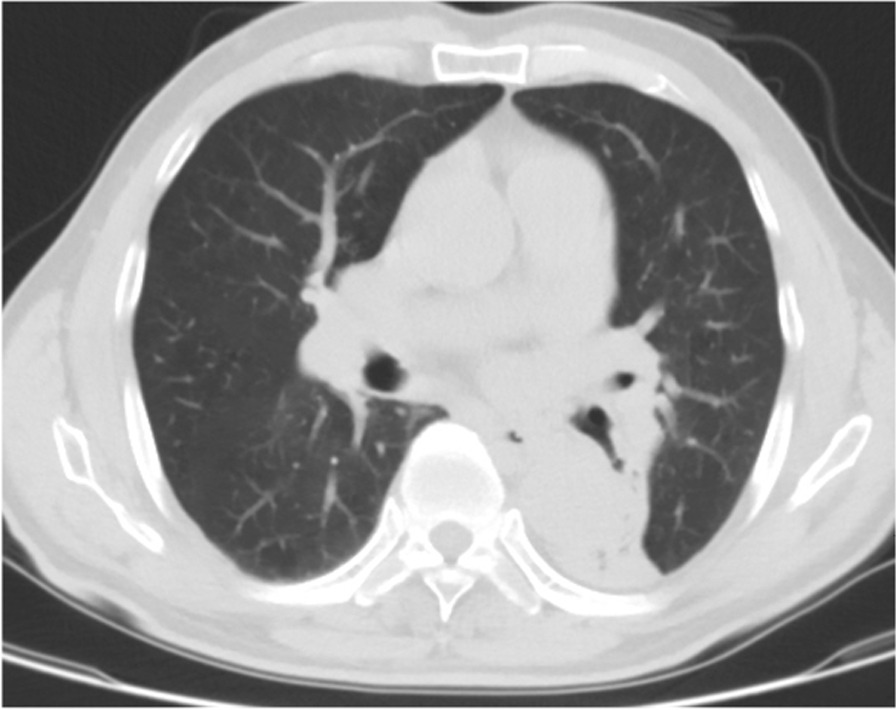
Chest computed tomography plain scan: left lower lobe bronchus partially occluded, left lower lobe consolidation

**Fig. 2:**
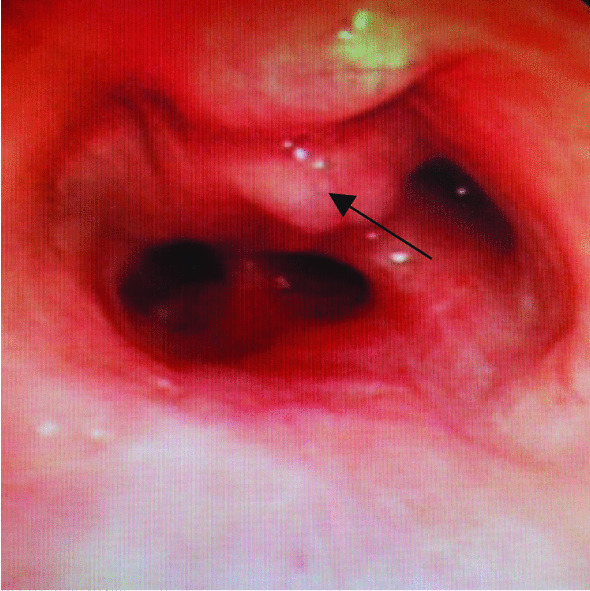
Bronchoscopy showed no pulsatile, smooth nodular on the surface of the basal segment of the left lower lobe. The mucosa was intact with mild congestion. Local hemorrhage was seen on the surface of the nodule by biopsy forceps. As shown in the figure, the abnormal blood vessel have been marked with arrows

**Fig. 3, 4:**
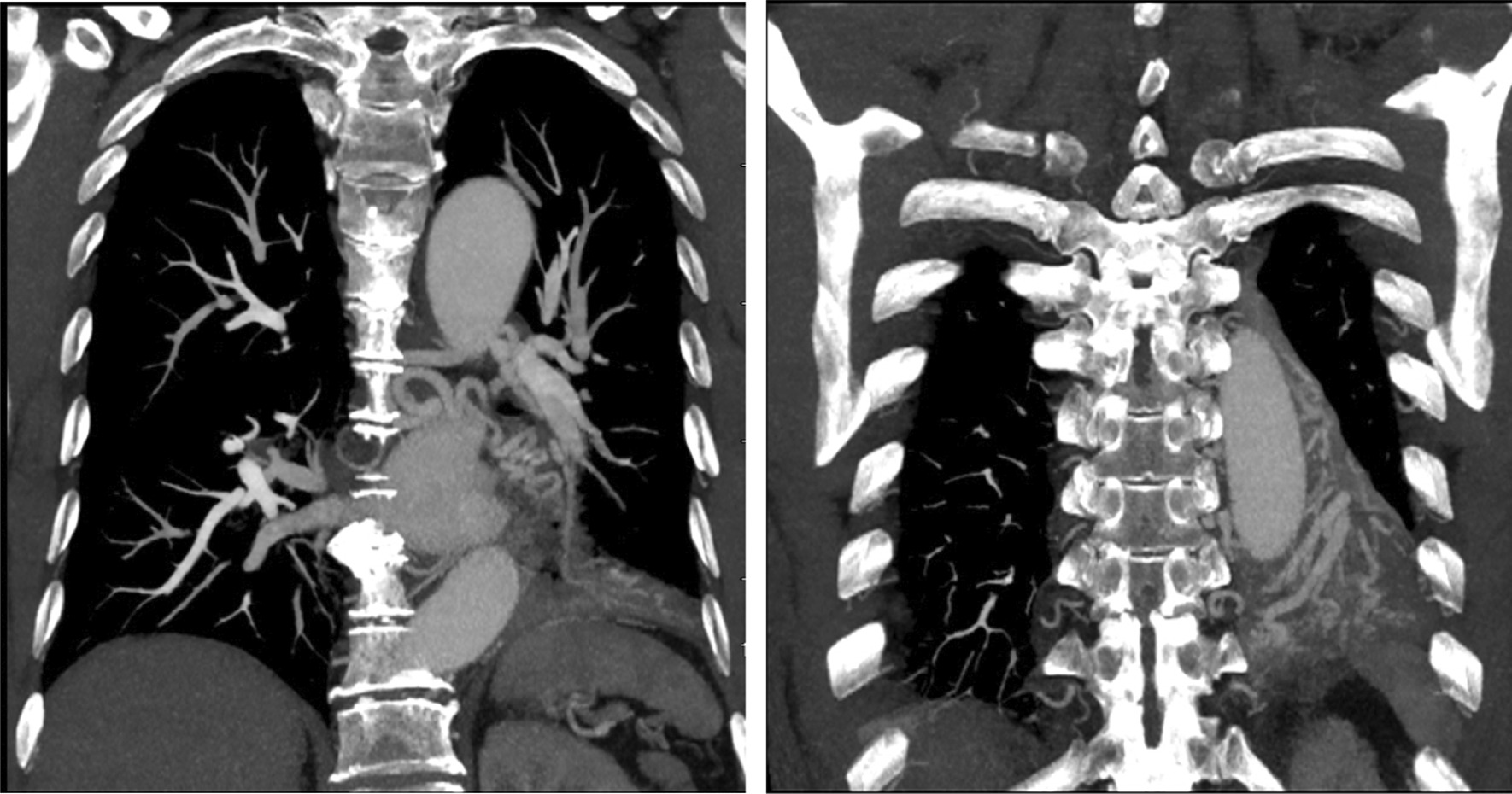
Computed tomography angiophy of bronchial artery (coronary plane): tortuous and dilated branches of left bronchial artery

**Fig. 5:**
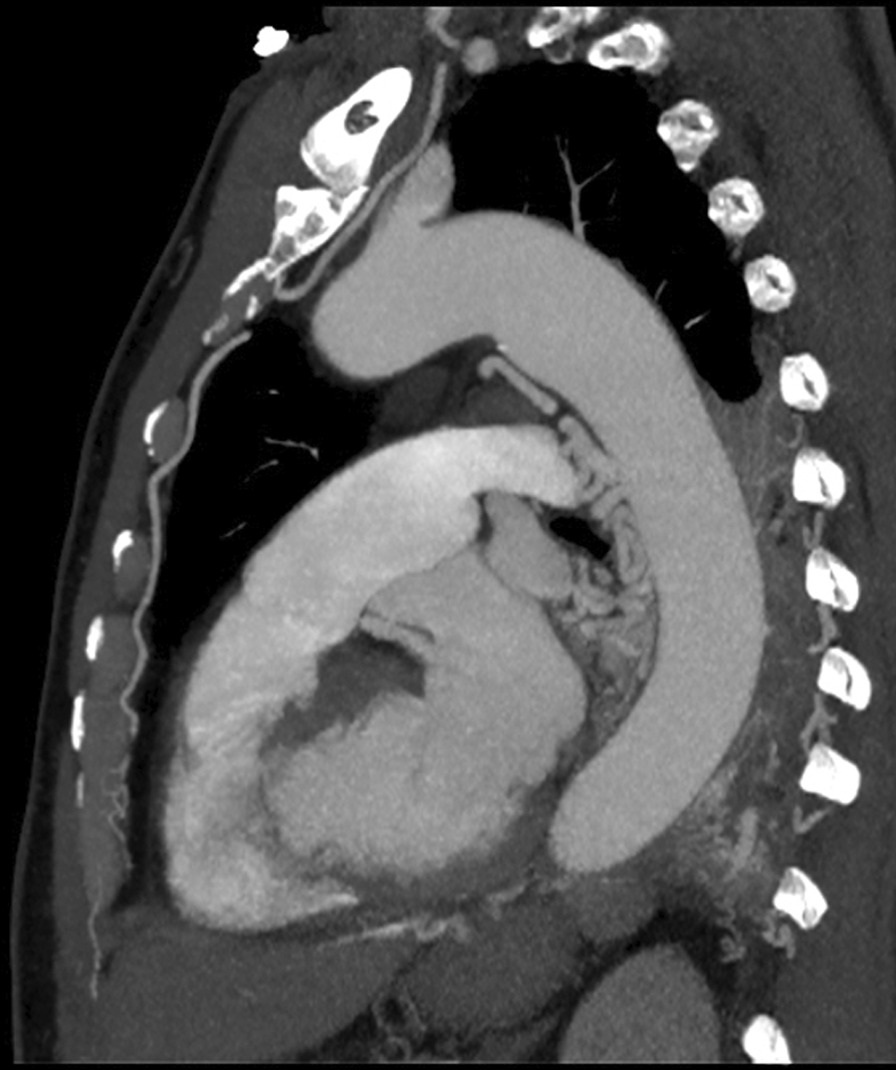
Computed tomography angiophy of bronchial artery (sagittal plane): tortuous and dilated branches of left bronchial artery

**Fig.6, 7:**
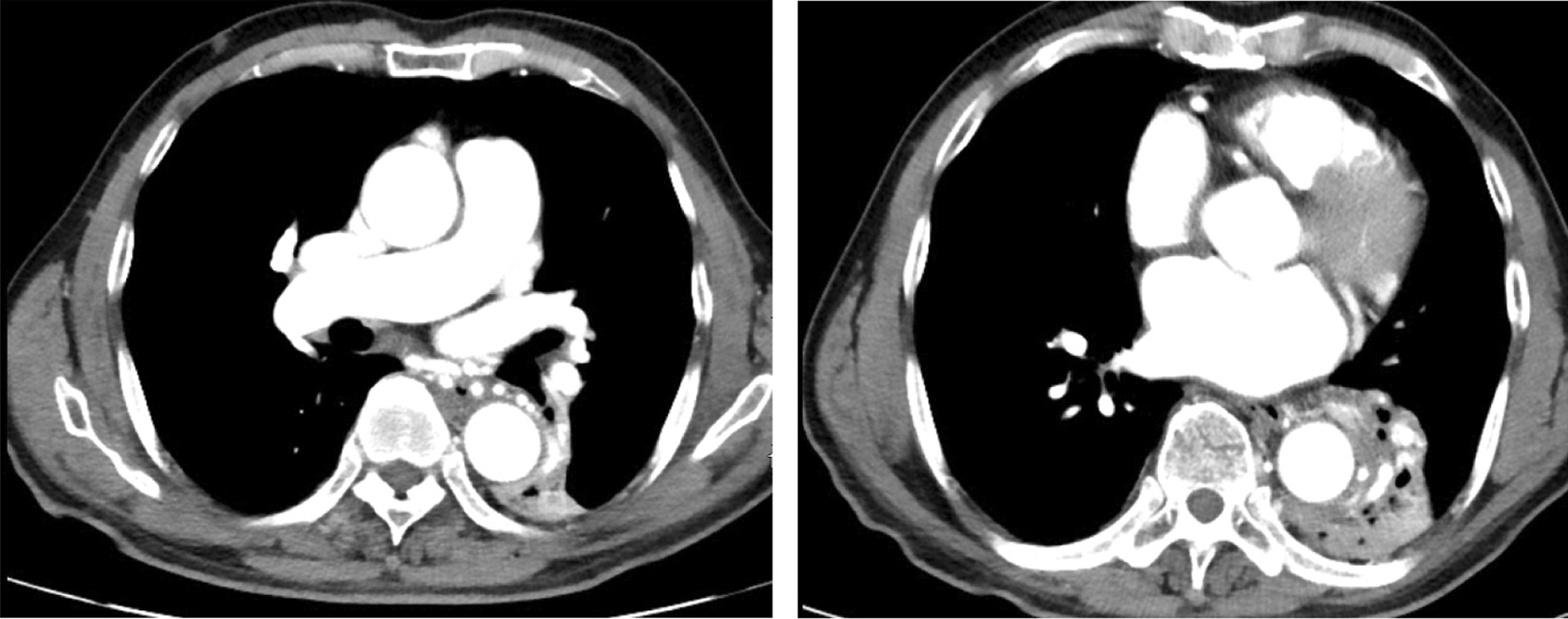
Computed tomography angiophy of bronchial artery (cross section): the left bronchial artery branches are tortuous and dilated, the volume of the left lower lobe of the lung shrinks, and the lung tissue consolidation shows soft tissue density shadow

**Fig. 8, 9:**
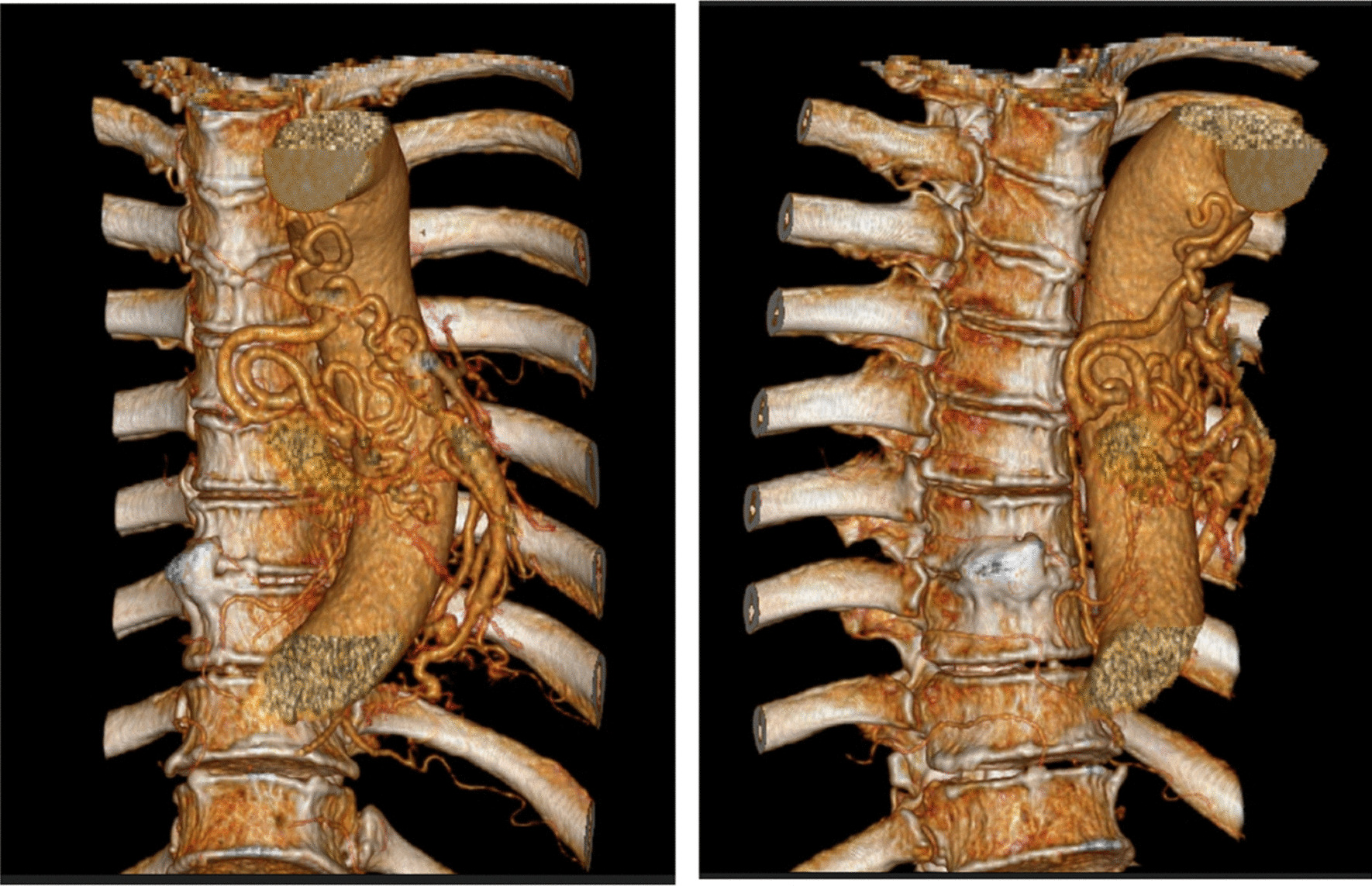
Computed tomography angiophy of bronchial artery (iterarive reconstruction): tortuous and dilated branches of left bronchial artery

**Fig. 10:**
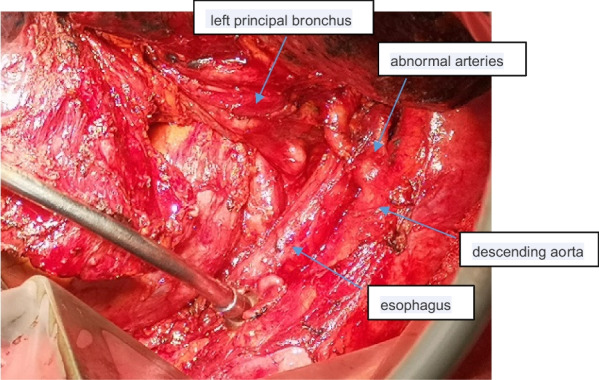
Surgical specimen: black arrow indicates abnormal twisted and thickened esophageal nutrient vessels and bronchial arteries from the descending aorta

A 68 year old male smoking for 30 pack-year was admitted to the hospital on December 5, 2018 due to "left lung shadow was found for 4 months, and cough with left subcostal pain for 2 months". After admission, chest enhanced CT showed that there was a leaf like solid density increase shadow in the lower left lung with obvious enhancement, and the left lower lobe bronchus was partially occluded (Fig. 1). Tracheoscopy showed that there were nodular processes at the opening of the basal segment of the left lower lobe, with smooth surface and complete mucosa (Fig. 2). Local bleeding was seen on the surface of the node at the opening of the basal segment of the left lower lobe touched by the biopsy forceps, with the amount of 100 ml. After immediate intratracheal injection of hemocoagulase 2 IU, adrenaline 1 IU and intravenous drip of pituitrin 6 IU, the bleeding gradually decreased. The next day, only a small amount of dark red blood filaments were found in the sputum. The bronchial artery CTA showed that the anterior wall of the thoracic aorta sent out the left and right bronchial arteries from the level of the 5th and 6th thoracic vertebrae respectively. The branches of the left bronchial artery were tortuous and dilated (Figs. 3, 4, 5, 6, 7, 8 and 9). However, two days later, the patient coughed violently at night and had massive hemoptysis. The total amount of hemoptysis was about 500 ml within half an hour. After hemostasis with hemocoagulase 2 IU and pituitrin 12 IU, the amount of hemoptysis gradually decreased. After consultation with the thoracic surgeon, it was decided that because of the large amount of bleeding and the clear cause of the disease, the possibility of massive bleeding again after medical treatment was large, the emergency left lower lobe resection was performed immediately. The operation chosen to enter the chest cavity through the posterolateral incision of the left chest. Extensive adhesion was found in the chest cavity, which was carefully separated by ultrasonic knife The exploration found that the left lower lobe of the lung was consolidated and could not be re-expanded. The left lower pulmonary vein was thin. There were tortuous and thickened arterial vessels on the surface of the left main bronchus and the lower lobe bronchus. The abnormal vessels originated from the aortic arch (Fig. 10), and were cut off at the root of the vessels with linear cut stapler. First, cut off the lower lobe pulmonary vein with linear cut stapler, fully expose the lower lobe bronchus, and cut off with linear cut stapler. Continue to dissociate the lower lobe pulmonary artery and cut it with linear cut stapler. Cut along the gap between the upper and lower lobes with linear cut stapler, and remove the surgical specimen. The procedure went smoothly, approximately 400 ml of blood was lost, and no transfusion was given. The operation lasted three and a half hours. The patient was discharged after 1 week in hospital. Postoperative pathology: alveolar atrophy in the "lower lobe of left lung", bronchiole dilatation and bleeding, muscular vascular wall uneven thickness, congestion, interstitial fibrous tissue hyperplasia, chronic inflammatory cell infiltration; One lymph node in "Group 5", seven lymph nodes under "carina" showed reactive hyperplasia, and "bronchial artery in mediastinum" showed thick walled vessels with uneven wall thickness. No new acute hemoptysis was observed after discharge, and the patient was still being followed up at the time of writing this report.

## Discussion

Massive hemoptysis is a common critical disease in respiratory medicine. The common causes include bronchiectasis, pulmonary tuberculosis or lung tumor. BDD is one of the rare causes of massive hemoptysis. Since Sweerts [2] and others first reported BDD in 1995, nearly 100 cases have been reported, mostly cases. We reported a case of BDD. In addition, we searched the databases of Pubmed, Web of Science, CNKI and Wanfang for "bronchial Dieulafoy disease". The database identified 65 articles from January 1995 to December 2021. After removing repeated reports, meetings, incomplete information and nursing literature, 60 articles were included, and 88 cases of BDD [2–61] were reported (Table 1).

**Table 1:** Basic Information of Patients (*N* = 88)

Basic information of the patient	Number of cases	Proportion (%)
*Gender*		
Male	54	61.4
Female	34	38.6
*Age*		
0–15	9	10.2
15–30	13	14.8
30–45	17	19.3
45–60	17	19.3
60–75	29	33.0
75–90	3	3.4
*Smoking history*		
Yes	36	40.9
No	31	35.2
Unknown	21	23.9
*Previous respiratory diseases*		
Tuberculosis	12	13.6
Chronic obstructive pulmonary disease	9	10.2
Bronchiectasis	6	6.8
Repeated infection (upper respiratory tract and lung)	8	9.1
Asthma	2	2.3
Pulmonary hypertension	1	1.1
*Clinical manifestation*		
Hemoptysis	73	83.0
Cough	17	19.3
Fever	4	4.5
Repeated pulmonary infection	4	4.5
Dyspnea or respiratory failure	10	11.4
Chest pain	1	1.1

There are 54 males and 34 females in 88 BDD patients, with a male to female ratio of 1.6:1. The minimum age of the patients is 9 months + 3 days, the maximum age is 85 years old, and the average age is 46.1 years old. All age groups have diseases, and about 1/3 of the patients are aged 60–75 years.

The etiology and pathogenesis of BDD remain to be clarified. At present, the possible mechanisms include: (1) congenital bronchial artery dysplasia [34]; (2) Chronic airway inflammation or injury [34]; (3) It is related to long-term heavy smoking [14]; (4) Acquired disease or normal vascular variation [27], which is easily misdiagnosed as bronchiectasis and other diseases in clinic. The 68 year old male patient we reported had a clear smoking history. In addition, 43.1% of the 88 patients were previously associated with other respiratory diseases. BDD may be related to chronic airway inflammatory injury or bronchial artery stretching and dilation caused by pulmonary tuberculosis [3, 6–8, 14, 20, 29, 33, 39, 42, 48], pneumonia [5, 12, 23, 24] and bronchiectasis [5, 16, 23, 27, 52, 57].

The most common symptom of BDD is repeated hemoptysis. It is reported that the maximum hemoptysis volume is 2000 ml [15]. However, the clinical manifestations of the disease are not specific, and patients may seek medical advice due to cough [23, 29, 45, 57], infection [16, 24, 45] or respiratory failure [3, 41, 43, 46]. Liu Yanhong and others once reported that a BDD patient only presented with chest pain but no hemoptysis. Later, hemoptysis occurred after bronchoscopic biopsy and was definitely diagnosed by bronchial angiography [27]. The patient we reported only presented with cough and left chest pain without hemoptysis. Therefore, if the patient has repeated massive hemoptysis of unknown causes, BDD should be considered; we also need to focus on patients with recurrent respiratory symptoms.

Auxiliary examinations of BDD include chest X-ray and computed tomography (CT), bronchoscopy and bronchial angiography. 36 patients received chest X-ray [2, 3, 6–8, 10–17, 19, 24, 30, 32, 33, 36, 39, 41–43, 49, 56, 60]. 78 patients received chest CT [2, 4, 5, 7–12, 14–20, 22–37, 39–49, 51–60]. The imaging manifestations are mainly inflammatory changes and ground glass shadows. Others include atelectasis, consolidation, bronchiectasis, nodules or masses or cavity in the bronchial, etc. (Table 2). The imaging findings of this patient reported here are mainly lung consolidation and atelectasis; Due to lack of specificity and sensitivity, it is not the first choice for diagnosis of BDD. 82 patients underwent bronchoscopy, of which 26 patients underwent bronchoscopic biopsy [3, 5, 8, 10–12, 15–18, 20, 23, 24, 29, 36–38, 45, 48, 49, 52, 57, 61], and 18 patients (22%) had severe hemoptysis complications [3, 5, 10, 16–18, 20, 23, 24, 29, 36, 37, 45, 48, 49, 52, 57, 61], and 10 patients died (12.2%) (Table 3) [3, 5, 16, 23, 24, 29, 45, 49, 52, 61]; Microscopically, the main manifestations were small (about 2–7 mm in diameter), no pulsatile, smooth nodules or mucosal protrusions, white caps on the surface, and some nodules may appear with active bleeding and blood clots in the bronchus; In some cases, the abnormal blood vessels in the submucosa present twisting and earthworm like dilation, sometimes presenting purple nodules [27]. Because of intra bronchial hemorrhage and blood clots, it is difficult to find small mucosal protrusions, or the mucosal protrusions are located below the subsegmental bronchus and cannot be found through conventional bronchoscopy. At this time, bronchial angiography can show the rich blood supply of the corresponding part of the lesion [25], the distortion, expansion, deformation of the bronchial artery and the bronchopulmonary fistula, which is helpful for the diagnosis of BDD (Fig. 3). In this case, the patient suffered from bleeding without clamping under fiberoptic bronchoscope, indicating obvious vascular defects. Endobronchial ultrasound (EBUS) is a new diagnostic method, which can determine the nature of bronchial mucosal protrusion, mainly manifested as an echo free area of submucosal lesions. Doppler mode can detect blood flow signals [18]. Nevertheless, the diagnosis still depends on pathology. The main pathological features of the disease are submucous bronchial artery expansion or abnormal artery rupture and bleeding [3]. Twisted, dilated and deformed arteries form small nodules with a diameter of several millimeters that protrude from the lumen of the bronchi and cover the bronchial mucosa; In some cases, the diseased bronchi are surrounded by abundant blood vessels, and part of them invade the bronchial wall and directly reach the submucosa [9]. The diagnosis of BDD is mainly based on the bronchoscopy, bronchial angiography and pathological findings of surgical or autopsy specimens. However, due to the risk of fatal bleeding, the necessity of pathological diagnosis remains controversial. In some cases, the diagnosis is based on the findings of bronchoscopy and bronchial angiography [6, 21, 22, 27, 33, 37, 42, 47, 51, 53].

**Table 2:** Chest CT findings (*N* = 78)

Chest imaging findings	Number of cases	Proportion (%)
Ground glass change	23	29.5
Inflammatory changes	34	43.6
Atelectasis	10	12.8
Consolidation	5	6.4
Bronchiectasis	10	12.8
Nodules or masses in the bronchial	6	7.7
Cavity	3	3.8
Negative	9	11.5

**Table 3:** Tracheoscopy findings (*N* = 82)

Tracheoscopy findings (*N* = 82)	Number of cases	Proportion (%)
Only blood clot or thrombus	16	19.5
Active bleeding or bleeding point	6	7.3
Nodular or prominent lesions	42	51.2
Non pulsatile processes	7	8.5
only white cap	3	3.7
Normal	8	9.8
Tracheoscopy biopsy (*N* = 26)	26	31.7
Massive bleeding	18	22.0
Death	10	12.2

Summarizing the lesions of BDD, we found that there were 62 cases of right bronchus (3 cases of right main bronchus, 15 cases of right upper lobe, 20 cases of right middle lobe, 24 cases of right lower lobe, 6 cases of right middle bronchus); 25 cases of left bronchus (5 cases of left main bronchus, 10 cases of left upper lobe, 10 cases of left lower lobe); There were 5 cases of bilateral bronchi and 1 case near the carina (Table 4). To sum up, BDD usually occurs in the right bronchus, accounting for about two thirds of the total cases. It is more likely to occur in the right bronchus, which may be related to its anatomical structure. The risk of abnormality of the right bronchial artery is higher due to the diversity of embryonic development of the right bronchial artery, which is a congenital etiology [4]. Therefore, for patients with hemoptysis of unknown cause, if bronchoscopy shows lesions similar to BDD and the lesions are located in the right bronchus, it is necessary to doubt whether there is abnormal development of bronchial artery, and biopsy should be avoided or careful to prevent massive hemoptysis. Most of the abnormal arteries originate from the bronchial artery system and a small part from the pulmonary artery [2].

**Table 4:** BDD lesion location and vascular origin (*N* = 88)

BDD lesion location and vascular origin	Number of cases	Proportion (%)
Left bronchus	25	28.4
Left main bronchus	5	5.6
Left upper lobe bronchus	10	11.4
Left lower lobe bronchu	10	11.4
Right bronchus	62	70.5
Right main bronchus	3	3.4
Right upper lobe bronchus	15	17.0
Right middle lobe bronchus	20	22.7
Right lower lobe bronchus	24	27.3
Right middle bronchus	6	6.8
Bilateral bronchus	5	5.7
Near the bulge	1	1.1
Abnormal blood vessel source		
Source bronchial artery system	67	76.1
Source pulmonary artery system	7	8.0
Unknown	14	15.9

The main treatment methods of BDD include conservative medical treatment, selective bronchial artery embolization (SBAE), lobectomy and argon plasma coagulation via bronchoscope. At present, SBAE is the preferred surgical method. For SABE or recurrent hemoptysis after embolization, lobectomy of the diseased lung is used. Eighty eight patients with BDD were summarized, including: (1) conservative drug therapy (9 cases); (2) Endobronchial intervention (7 cases); (3) Bronchoscopic arterial embolization only (30 cases); (4) Pulmonary lobectomy only (15 cases); (5) Bronchial artery embolism + pulmonary lobectomy (24 cases) (Table 5). Two patients were successfully treated with argon plasma coagulation through bronchoscope [28, 39]. One patient failed to receive cryotherapy, and then placed silica gel stent [13]. 54 patients tried first-line selective embolization, and 24 patients received lobectomy due to unsuccessful embolization or prevention of bleeding. 66 patients were followed up for improvement without hemoptysis; Ten patients died (all of them were caused by massive hemorrhage during bronchoscopic biopsy). SBAE is usually used as the first-line method to treat hemoptysis, which is effective for some patients; The reported cases of intercostal artery embolism failure partially confirmed that the abnormal blood vessels originated from the pulmonary artery, and pulmonary lobectomy was necessary to control the bleeding.

**Table 5:** BDD treatment plan (*N* = 88)

Treatment plan	Number of cases	Proportion (%)
Conservative treatment	9	10.2
Bronchial Artery Embolization Only	30	34.1
Lobectomy only	15	17.0
Bronchial artery embolism (failure) + lobectomy	15	17.0
Bronchial artery embolism (successful) + lobectomy	9	10.2
Intervention under tracheoscope		
Freeze + place dumon silica gel bracket	1	1.1
Argon ion coagulation (apc)	2	2.3
Endotracheal intubation + mechanical ventilation	4	4.5
Untreated	3	3.4

In conclusion, the case we reported and the 88 cases we summarized support that BDD should be considered when recurrent and unexplained hemoptysis occurs and the lesion is limited to the right bronchus. Bronchial biopsy should be avoided and bronchial arteriography should be performed as early as possible. SBAE can be used for stable or intolerant patients to reduce the risk of life-threatening hemoptysis. Although it can retain some functions of the diseased lung, it is easy to recur after treatment. For uncontrollable cases or cases with poor prognosis, lobectomy can be the best choice to save lives. Although it will affect the quality of life of patients, it can eliminate the possibility of recurrence and obtain histopathological confirmation.

## Data Availability

The data that support the findings of this study are available from the corresponding author, Bing Li & Xuewei Zhao, upon reasonable request.
